# Topical Nonsteroidal Anti-Inflammatory Drugs for Macular Edema

**DOI:** 10.1155/2013/476525

**Published:** 2013-10-21

**Authors:** Andrea Russo, Ciro Costagliola, Luisa Delcassi, Francesco Parmeggiani, Mario R. Romano, Roberto dell'Omo, Francesco Semeraro

**Affiliations:** ^1^Eye Clinic, Department of Neurological and Vision Sciences, University of Brescia, Piazzale Spedale Civili 1, 25123 Brescia, Italy; ^2^Eye Clinic, Department of Health Sciences, University of Molise, Via de Santis, 86100 Campobasso, Italy; ^3^Department of Ophthalmology, University of Ferrara, Corso Giovecca 203, 44121 Ferrara, Italy; ^4^Eye Clinic, Istituto Clinico Humanitas, Via Manzoni 56, 20089 Milano, Italy

## Abstract

Nonsteroidal anti-inflammatory drugs (NSAIDs) are nowadays widely used in ophthalmology to reduce eye inflammation, pain, and cystoid macular edema associated with cataract surgery. Recently, new topical NSAIDs have been approved for topical ophthalmic use, allowing for greater drug penetration into the vitreous. Hence, new therapeutic effects can be achieved, such as reduction of exudation secondary to age-related macular degeneration or diabetic maculopathy. We provide an updated review on the clinical use of NSAIDs for retinal diseases, with a focus on the potential future applications.

## 1. Introduction

Nonsteroidal anti-inflammatory drugs (NSAIDs) are one of the most commonly prescribed classes of medication, and they are routinely employed for their analgesic, antipyretic, and antiinflammatory properties. Because they are potent inhibitors of cyclooxygenase (COX) enzymes, they reduce the synthesis of pro-inflammatory prostaglandins (PGs). NSAIDs have been widely used systemically for many decades and have more recently become available in the form of topical ophthalmic formulations [1]. In ophthalmology, topical NSAIDs are mostly used to stabilize pupillary dilation during intraocular surgery, to control postoperative pain and inflammation (particularly after refractive surgery), and to treat allergic conjunctivitis and pseudophakic cystoid macular edema (CME) [2, 3]. A growing body of evidence suggests that NSAIDs may also be beneficial in diabetic retinopathy (DR), ocular tumors, and age-related macular degeneration [1, 4–8]. This review focuses on the potential application of NSAIDs to treat retinal disease. 

## 2. NSAIDs and Cyclooxygenases

COX enzymes are an active component of the inflammatory process. They catalyze the biosynthesis of eicosanoids from arachidonic acid to produce 5 classes of PGs: PGE_2_, PGD_2_, PGF_2*α*_, PGI_2_, and thromboxane A_2_ [1]. Ocular actions of PGs are manifested in three ways [9]. Firstly, they act on intraocular pressure (IOP). PGE_2_ increases the IOP by local vasodilation and increased permeability of blood aqueous barrier. On the other hand PGF_2*α*_ lowers the IOP, which is attributed to increased uveoscleral outflow. Secondly it acts on iris smooth muscle to cause miosis. Thirdly, PGs cause vasodilation and increase the vascular permeability with the disruption of the blood-ocular barrier with leukocyte migration and therefore edema formation [10]. By definition, NSAIDs lack a steroid nucleus.

COX-1 and COX-2 are the main COX isoforms, although there is a third isoform, COX-3. COX-3 is an acetaminophen-sensitive alternatively spliced variant of COX-1, and it has not been well defined [11–13]. COX-1 regulates normal physiological processes and is mainly expressed in the gastrointestinal tract, kidneys, platelets, and vascular endothelium. COX-2 is the predominant isoform in the retinal pigment epithelium (RPE) [14] and is upregulated during inflammatory processes, pain, and fever, but it is also expressed under normal conditions in sites such as the brain and kidneys [15]. COX-2 has also been found in choroidal neovascularization (CNV) and in DR [4, 5, 7, 8, 16–19]. PGs act by upregulating a number of soluble mediators responsible for the expression of vascular endothelial growth factor (VEGF), which plays a key role in the CNV and in the DR [20–22]. In a number of experimental models COX-2 inhibition has been found to inhibit angiogenesis [23–26], CNV, and DR [17, 18, 27, 28].

*Commercially Available Formulations*. NSAIDs are a chemically heterogeneous group of molecules, described in detail elsewhere [29]. There are six major classes: salicylates, indole acetic acid derivatives, enolic acid derivatives, fenamates, aryl acetic acid derivatives, and aryl propionic acid derivatives. However, the topical NSAIDs available for ophthalmic usage are mostly limited to the soluble forms: indole acetic, aryl acetic, and aryl propionic acid derivatives [9, 16]. A list of commercially available NSAID eyedrops is provided in Table 1.

Most of the NSAIDs are weakly acidic drugs, which ionize at the pH of the lacrimal fluid and therefore have limited permeability through the anionic cornea which has an isoelectric point (pI) of 3.2 [9]. Reducing the pH of the formulation increases the unionized fraction of the drug which enhances permeation. Because of their acidic nature, NSAIDs are inherently irritating [30]; reducing the pH further increases their irritability and decreases their aqueous solubility. In addition, the anionic nature of NSAIDs leads to the formation of insoluble complexes with cationic quaternary ammonium preservatives, such as benzalkonium chloride [31]. Hence, a NSAID formulation that is comfortable when topically applied is somewhat difficult to formulate.

## 3. Pharmacokinetics and Pharmacodynamics

NSAIDs are adsorbed by the gastrointestinal tract, reaching a peak serum concentration after 1–3 hours. They are metabolized by the liver and excreted in the urine and bile; they are highly protein bound in the plasma (>95%), normally to albumin; thus their volume of distribution approaches that of plasma [16]. Topically administered NSAIDs follow this distribution, since they are systemically adsorbed by the nasolacrimal outflow system and the mucosal surfaces.

Nepafenac is a prodrug that is rapidly converted to the more potent amfenac by intraocular hydrolases. Since nepafenac is a noncharged molecule, it exhibits greater corneal permeability than the other NSAIDs. This was demonstrated in an *in vitro* study that showed sixfold greater corneal penetration by nepafenac than by diclofenac [34]. Bromfenac has a similar structure to amfenac, with the exception of a bromine atom at the C_4_ position. This modification increases the penetration of bromfenac into ocular tissues, increasing its anti-inflammatory activity.

Ketorolac is reportedly the most potent inhibitor of COX-1, while bromfenac and nepafenac/amfenac are the most potent inhibitors of COX-2 [9, 35, 36]. However, ketorolac 0.45% inhibited PGE_2_ more strongly than bromfenac 0.09% and nepafenac 0.1%, reaching significantly greater aqueous concentrations [37, 38]. Bromfenac has been reported to be a 3- to 18-fold more potent inhibitor of COX-2 than diclofenac, ketorolac, and nepafenac/amfenac, although these data remain to be confirmed in randomized controlled clinical trials [1, 9, 39]. It is possible that COX-1 may also play a role in inflammation [1, 16] therefore the specific roles of COX-1 and COX-2 in this context require further investigation.

A number of studies have measured intraocular NSAID levels after topical administration. After a single eye-drop, peak aqueous drug levels are detectable for diclofenac 0.1% (82 ng/mL; 2.4 h peak), flurbiprofen 0.03% (60 ng/mL; 2.0 h peak), nepafenac 0.1% (205.3 ng/mL; 30 min peak), amfenac (following administration of the prodrug nepafenac 0.1%; 70.1 ng/mL), ketorolac 0.4% (57.5 ng/mL; 60 min peak), and bromfenac 0.09% (25.9 ng/mL) [35, 40]. 

More prolonged and more frequent administration of NSAIDs leads to higher aqueous levels. Twelve doses of ketorolac 0.4% over 2 days reportedly result in an aqueous level of 1079 ng/mL, and the same dosing regimen of nepafenac 0.1% results in 353 ng/mL; both concentrations far exceed that is reportedly required to inhibit COX-1 and COX-2, which is 50 ng/mL [41].

While topical administration of NSAIDs achieves therapeutic levels in the aqueous humor, thereby reducing the synthesis of PGs in the ciliary body and the iris, such a therapeutic effect is less evident in the retina and the choroid. Few studies have measured NSAID levels in the human vitreous cavity after topical administration. Heier et al. [42]. measured vitreous drug levels in patients who received ketorolac 0.4% QID, bromfenac 0.09% BID, or nepafenac 0.1% TID for 3 days before vitrectomy. Vitreous levels of ketorolac, bromfenac, and amfenac were reportedly 2.8 ng/mL, 0.96 ng/mL, and 2.0 ng/mL, respectively, but only ketorolac resulted in significantly lower vitreous PGE_2_ levels compared to placebo.

NSAIDs inhibit the expression of COX enzymes, thereby reducing the endogenous PGs that act on the iris and ciliary body to induce vasodilation, blood-ocular barrier disruption, leukocyte migration, pain stimulation, IOP control, and miosis [2, 3, 16, 43]. Commercially available PGF_2*α*_ analogues act by increasing uveoscleral outflow in the ciliary body, while PGE_2_ reportedly increases IOP via vasodilation and partial disruption of the blood-ocular barrier [43]. The administration of topical NSAIDs does not have any effect on IOP, as it is not selective with regard to PG class. However, NSAIDs may have a slight additive effect when administered together with PGF_2*α*_ analogous [44, 45]. A pivotal difference between NSAIDs and corticosteroids is the effect of the latter on both IOP and lipoxygenase, which facilitates a greater anti-inflammatory effect, albeit with an associated increase in the likelihood of IOP elevation. 

## 4. Macular Edema after Cataract Surgery

There is convincing clinical evidence in the peer-reviewed literature attesting to the capacity of topical NSAIDs to reduce postoperative inflammation after eye surgery [1, 2, 16, 46]. In randomized controlled clinical trials, bromfenac 0.09%, nepafenac 0.1%, diclofenac 0.1%, ketorolac 0.5%, flurbiprofen 0.03%, and indomethacin 1% have been shown to decrease postoperative inflammation following cataract surgery [34, 41, 47–58]. Corticosteroids are also widely used postoperatively to reduce inflammation; therefore studies comparing the 2 drug classes have been conducted. While significant differences in the reduction of intraocular inflammation after cataract surgery were not observed [55, 56, 59], NSAIDs were more effective at reestablishing the blood-aqueous barrier as indicated by flare, which was measured via either slit-lamp examination or fluorophotometry [16, 46, 52, 59]. Thus, the collective evidence suggests that topical NSAIDs may be used in place of topical corticosteroids after cataract surgery or, perhaps preferably, in addition to them; a number of clinical trials have reported a synergistic effect when NSAIDs and corticosteroids are administered together [49, 50, 58, 60, 61].

Despite advances in technique and surgical materials, cystoid macular edema (CME) is the most frequent cause of reduced vision following uneventful modern cataract surgery, with a seemingly rare incidence of 0.1–2.35% for clinically significant CME [62–64]. Also known as Irvine-Gass syndrome, it is mainly caused by the accumulation of extracellular fluid within the retina due to leakage from dilated capillaries [1, 16, 63]. The pathogenesis of it is not fully understood, but the main trigger is thought to be surgical trauma of the intraocular tissues, involving rupture of the blood-aqueous barrier; this may cause diffusion of PGs and other inflammatory mediators into the vitreous cavity, inducing a cascade of inflammatory events with subsequent rupture of the blood-retinal barrier, resulting in CME in some patients [64]. Therefore it seems reasonable to take strong measures to minimize the inflammatory process, possibly including the administration of both corticosteroids and NSAIDs together. A recent study by Ersoy et al. [65] that quantitatively assessed aqueous flare after phacoemulsification reported that patients who developed CME had significantly higher flare values than those who did not, suggesting that inflammatory pathogenesis and a breakdown of the blood-ocular barrier may be involved.

CME can be diagnosed and classified clinically, on fluorescein angiography and by optical coherence tomography (OCT). The range of the reported incidence rates is wide (0.10–2.35% for clinically important CME, defined as a retinal thickening within 500 microns of the center of the macula causing a significant vision impairment) [62, 64], which may be due to the different patient populations, cataract stages, surgical techniques, and, particularly, diagnostic methods utilized by the relevant studies. Notably, after small-incision cataract surgery the reported rates of CME range from 9 to 19% based on fluorescein angiography and are as high as 41% as determined by OCT [66–68], although clinically important CME is far less common [1, 69]. 

A number of studies report the effectiveness of topical NSAIDs in the prophylaxis of CME following cataract surgery [2, 16, 63, 70–74], although the angiographic reduction of CME is reportedly most evident in the first postoperative month and is no longer statistically significant a year after surgery. However, interpretation of the independent effects of NSAIDs based on the results of the available studies is difficult, due to the common concomitant administration of corticosteroids. One trial by Flach et al. [73] reported that prophylactic use of ketorolac 0.5% was effective in reducing CME without the use of corticosteroids. Miyake et al. [75] prospectively compared the effects of topical diclofenac 0.1% versus fluorometholone 0.1% (a corticosteroid with limited intraocular penetration that therefore could be reasonably approximated to a placebo) in the prophylaxis of CME and reported that 5 weeks after surgery, angiographic CME was present in 5.7% of diclofenac-treated eyes and 54.7% of fluorometholone-treated eyes. 

A randomized comparison of topical ketorolac 0.4% plus corticosteroid versus corticosteroid alone showed a significantly reduced rate of CME with combination treatment after phacoemulsification [58]. However, the incidence of definite or probable CME (definite CME is intended as the presence of cystoid changes associated with ≥40 *μ*m retinal thickening evident on OCT, while probable CME is intended as the presence of changes in retinal contour and increased macular thickness relative to preoperative baseline, but without definite cystoid changes) was low in both groups (2.4% in the corticosteroid group and 0% in the ketorolac/corticosteroid group) and there was no difference in visual outcomes. Such results raise the issue of the cost effectiveness of routine administration of CME prophylactic treatment with both corticosteroid and NSAIDs for patients at low risk of CME. However, cost effectiveness ratio is certainly lower in diabetic and uveitic patients who are at higher risk of CME and are reported to benefit from routine concomitant use of NSAIDs and corticosteroids [76].

CME following phacoemulsification may be treated early (less than 6 months) or late (6 months or more) following its diagnosis, respectively, defining acute and chronic CME [1, 16]. The treatment of chronic CME following cataract surgery has been assessed in a number of studies [16, 77–80] which have shown an overall beneficial effect of NSAID treatment, although a recent meta-analysis [81] reported that for acute CME, the evidence is not yet conclusive. This finding is consistent with a recent study by Almeida et al. [82]. assessing the efficacy of ketorolac and nepafenac with regard to the prevention of postoperative CME after uneventful phacoemulsification. The authors concluded that prophylactic topical NSAIDs are not recommended for routine surgery patients without risk factors.

In chronic disease, trials by Flach et al. [63, 79, 80] suggest that topical ketorolac 0.5% is effective and that treatment for a duration of 3 months provides a longer lasting benefit than treatment for 2 months. However, there are few published trials and they tend to have small sample sizes; therefore, further controlled studies are required.

Four topical NSAIDs (diclofenac 0.1%, ketorolac 0.4%, nepafenac 0.1%, and bromfenac 0.09%) have been evaluated in combination with intravitreal corticosteroid and bevacizumab injections for the treatment of chronic pseudophakic CME [83]. Results suggested that while NSAIDs apparently provide additional benefit to that produced by corticosteroids and anti-VEGF, only nepafenac- and bromfenac-treated eyes showed reduced retinal thickness at 12 and 16 weeks, and only nepafenac led to a significant improvement in vision. Similarly, in a retrospective and uncontrolled study, nepafenac 0.1% improved retinal thickness and visual acuity in patients with chronic CME [84].

In all reported studies NSAIDs are administered using the traditional BID, TID, and QID regimens. Further studies are needed to enlighten if varying the dosing regimen affects the efficacy of NSAIDs in CME resolution.

In conclusion, although there is no specific approved treatment for pseudophakic CME, overall evidence supports the administration of topical NSAIDs and also suggests that combining them with topical corticosteroids yields a synergistic effect. However, the advisability of NSAIDs for CME prophylaxis in patients with low risk factors is debatable, given the low clinically significant incidence and the cost effectiveness ratio. 

## 5. Age-Related Macular Degeneration

In developed countries, age-related macular degeneration (AMD) is the leading cause of visual impairment and blindness in patients over 60 years of age [85]. Typical features of neovascular AMD include choroidal neovascularization (CNV) beneath the macula, with associated retinal hemorrhages and swelling. Involution of the new vessels is accompanied by fibrous metaplasia, permanent loss of photoreceptors, and disciform scarring, which often result in loss of central vision [86]. Large-scale clinical trials have demonstrated that monthly intravitreal injection of anti-VEGF prevents vision loss and may even improve visual acuity in patients with neovascular AMD [87, 88]. 

VEGF is not the sole cause of CNV. Inflammation plays an important role and some patients exhibit an inadequate response to anti-VEGF treatment, along with persistent exudation [89]. In particular, a multitude of recent genetic analyses in human AMD patients supports the role of complement factor H in the pathogenesis of it in up to 50% of cases [90–93].

The complement system is a major contributor to innate immunity. There are several complement components (C3, C5, the C5b-9 membrane attack complex (MAC), and CD46) found in drusen. This indicates that complement components and regulators may contribute to the formation of drusen and upregulate VEGF expression [94–96]. Although AMD is not a classic inflammatory disease, inflammatory cells have an important role in AMD pathogenesis and progression [94, 97]. Autoimmunity has also been suggested to have a role in drusen formation and AMD pathogenesis. It has been suggested that the presence of a number of antiretinal autoantibodies such as anticarboxyethylpyrrole and antiastrocyte antibodies is an early feature of AMD pathogenesis [98, 99]. Recently, Morohoshi et al. [100] demonstrated that 94% of patients with early-stage AMD and 83% of patients with late-stage AMD had elevated levels of serum retinal autoantibodies, compared with only 9% of normal controls.

NSAIDs may have a protective effect with regard to Alzheimer's disease, reducing its prevalence [101, 102], and similarly a prospectively followed group of patients under long-term anti-inflammatory treatment for rheumatoid arthritis showed a very low prevalence of AMD [103]. Moreover, a larger retrospective study reported reduced rates of CNV among AMD patients undergoing aspirin treatment [104]. Even the anecdotal use of loxoprofen sodium for toe cellulitis has been reported to improve CNV [105]. However, a more recent Australian population-based study reported that regular aspirin use is associated with increased risk of incident neovascular AMD [106]. This is consistent with a report emerging from the European Eye Study [107] that frequent aspirin use is associated with early AMD and late wet AMD and the odds ratio rises with increasing frequency of consumption. Nevertheless, evidence supports the additive role of NSAIDs in the treatment of CNV, with a protective effect that is probably due to the control of both inflammation, and COX-2 which is a known promoter of angiogenesis and can be found in CNV [8, 19, 24, 108]. Pharmacological inhibition of COX seems to reduce VEGF expression in cultured human RPE cells [8, 109]. Kim et al. [17, 18] have demonstrated that both topical and intravitreal ketorolac significantly reduce angiographic leakage and retinal levels of PGE_2_ and VEGF in an animal model of CNV. 

Therefore, the addition of an anti-inflammatory agent could be a valid option for controlling CNV, as simply inhibiting VEGF addresses neither the multifactorial pathogenesis of CNV nor the underlying cause of VEGF production.

Although the evidence coming from human clinical trials is less consistent than that arising from animal models, a favorable effect of additive topical NSAID therapy with regard to anti-VEGF for the control of exudative AMD has recently been reported in 3 prospective, randomized, and controlled clinical studies (Table 2) [4, 5, 7].

Russo et al. [7] demonstrated that topical ketorolac acts in conjunction with intravitreal anti-VEGF treatment; central macular thickness (CMT) is significantly lower (−37.1 *μ*m) after 6 months in patients receiving combination therapy, although there were no differences in either visual acuity or the number of injections between the 2 groups. Such results are partially in contrast with the findings of Gomi et al. [5], in which the authors also reported a reduction in the frequency of ranibizumab injections over 6 months when topical bromfenac was used as an adjunctive treatment with ranibizumab. However, in addition to the differences in the pharmacological properties of bromfenac and ketorolac, another point of difference was that Gomi et al. [5] administered just one ranibizumab injection and then treated the patients on an as-needed basis; therefore the number of injections administered was not consistent. Similar results were reported in another recently published trial [4] evaluating the use of topical bromfenac in combination with ranibizumab versus ranibizumab alone. A significantly greater reduction in CMT was found after 12 months in the combination group (−28.3% versus −18.9%), without concomitant differences in visual acuity changes between the 2 arms [4].

Such findings are in contrast with 2 previous retrospective studies [32, 33] that did not detect any improvement in visual acuity or in CMT, with the addition of bromfenac or nepafenac in conjunction with anti-VEGF administration. However, these inconsistent results may be due to differences in study design (shorter retrospective design and smaller sample sizes) and the presence of recalcitrant and persistent exudative AMD in the examined cohorts, which render direct comparisons problematic.

Overall the literature supports the concomitant off-label administration of topical NSAIDs with on-label anti-VEGF intravitreal therapy, as NSAIDs act synergistically to reduce CMT in CNV. It will be important to evaluate the long-term efficacy of NSAIDs, as AMD is a chronic disorder. In particular, careful attention should be paid to the corneal complications associated with long-term use of topical NSAIDs.

## 6. Diabetic Retinopathy

DR is the most frequent cause of legal blindness in working-age individuals in developed countries [110]. In addition to DR, diabetic patients can suffer from diabetic macular edema (DME), which is caused by breakdown of the blood-retinal barrier resulting in leakage of plasma and water from small vessels [111]. These leakages result in swelling and thickening of the retina around the macula, the central part of the retina in which fine visual discrimination occurs. In patients with type 2 diabetes, DME is the primary cause of moderate and legal blindness [112].

Growing scientific evidence shows that an immunological cascade has a major role in the pathogenesis of DR [113]. Increased levels of inflammation mediators and PGs in DR have been found in the vitreous cavity in both animal and human studies [22, 114, 115], and the level of PGE_2_ correlates significantly with vitreous levels of VEGF [116]. The role of inflammation in the progression of DR has also been indirectly supported in a recent study [117] by the Diabetic Retinopathy Clinical Research Network, in which authors concluded that intravitreal triamcinolone appears to be associated with a reduced risk of worsening of proliferative DR.

In animal models PGs stimulate VEGF expression [17], and in cultured Muller cells agonism and antagonism of the PGE_2_ receptor increase and decreases VEGF production, respectively, in a dose-dependent manner [118]. In fact, NSAID treatments have been shown to prevent or delay DR progression in animal models [21, 27, 28, 119].

While no benefit was found in advanced DR in the Early Treatment Diabetic Retinopathy Study [120] examining the effect of 650 mg aspirin, the incidence of DR is reduced in human patients taking salicylates for rheumatoid arthritis [121], just as previously reported with exudative AMD, attesting to the contribution of COX to the development of DR. Such findings were confirmed in the Dipyridamole Aspirin Microangiopathy Diabetes Study (DAMAD) [122] that assessed the effect of 990 mg aspirin in early DR; a significant protective effect was associated with high doses of aspirin, which slowed the development of retinal microaneurisms. Subsequently, either 2 prospective randomized studies confirmed these findings with the administration of sulindac and celecoxib [123, 124].

The benefits of topical NSAID therapy for DR control are mainly reported anecdotally or in uncontrolled retrospective case studies. Pseudophakic DME showed improvement in retinal thickness and visual acuity after treatment with nepafenac 0.1% for 6 months in a case report [84]. Similarly, in a case series of 6 eyes with DME that were treated with nepafenac 0.1%, the average foveal thickness decreased significantly from 417 *μ*m to 267 *μ*m after a mean of 178 days. Authors moreover reported that four eyes gained vision and two eyes maintained vision, with a statistically significant mean visual acuity improvement from 0.78 logMAR to 0.67 logMAR [125]. Such results suggest that nepafenac 0.1% may exhibit activity against diabetic macular edema and warrant further investigation in larger, controlled studies, possibly with and without associated anti-VEGF therapy. In this regard a placebo-controlled study to assess the effect of nepafenac 0.1% on macular retinal volume in eyes with noncentral DME is being conducted (ClinicalTrials.gov Identifier: NCT01331005).

The intravitreal route is a privileged route for the delivery of drugs to the posterior eye, and it has been proposed as the route of administration for NSAIDs to treat DME. Evidence emerging from published case reports collectively suggests an increase in visual acuity without significant changes in the CMT. Soheilian et al. [126] evaluated the effect of a single dose of intravitreal diclofenac (500 *μ*g/0.1 mL) on 5 eyes with clinically significant diabetic macular edema and reported prominent improvements in visual acuity with no significant decrease in CMT. A similar result was reported by do Ceu Afonso Reis et al. [127] in a study involving 20 patients with DME refractory to retinal photocoagulation, who were treated with intravitreal ketorolac (500 *μ*g/0.1 mL) in one eye only. These findings are consistent with a study by Maldonado et al. [128] who treated 25 patients with ketorolac at a dose of 3000 *μ*g. On the other hand, Elbendary and Shahin [129] randomized 32 eyes in a 1 : 1 ratio to treatment with either 500 *μ*g/0.1 mL of diclofenac or 4 mg/0.1 mL of triamcinolone and reported a significant reduction in CMT with both treatments, but improvements in visual acuity were only evident in the triamcinolone group.

## 7. Conclusion

The initial pathological changes in macular edema appear in macular photoreceptors, RPE, Bruch's membrane, and choriocapillaris [97]. While their etiology is not fully understood, it is incontrovertible that inflammation has a critical role in the various manifestations of macular edema and its progression. The fact that inflammation is a common denominator in pseudophakic, exudative AMD and diabetic macular edema may explain why anti-inflammatory agents are beneficial as preventive or adjunctive therapies.

Considering our growing understanding of the underlying role of PGs, complement, and inflammation in eye diseases, the clinical use of topical NSAIDs will likely continue to expand. The newer and more potent topical formulations emerging are also likely to contribute to this expansion.

In summary, topical NSAIDs could be used alone for pseudophakic CME or as a favorable adjunct together with anti-VEGF for exudative AMD. Cost effectiveness ratio must be considered given the low incidence of pseudophakic CME in low-risk patients; however, the heavy economic burden of anti-VEGF injections that could potentially be reduced if future studies support the use of NSAIDs should also be considered.

## Figures and Tables

**Table 1:** Commercially available topical NSAIDs.

Molecule	Class	Administration
Indomethacin 0.5%	Indole acetic acid derivative	TID, QID
Ketorolac tromethamine 0.5%	Aryl acetic acid derivative	TID, QID
Bromfenac 0.09%	Aryl acetic acid derivative	BID
Nepafenac 0.1% (prodrug converted to amfenac)	Aryl acetic acid derivative	TID
Diclofenac 0.1%	Aryl acetic acid derivative	QID
Flurbiprofen 0.03%	Aryl propionic acid derivatives	QID
Pranoprofen 0.1%	Aryl propionic acid derivatives	TID
Piroxicam 0.5%	Enolic acid derivatives	TID, QID

BID: 2 times a day; TID: 3 times a day; QID: 4 times a day.

**Table 2:** Studies investigating NSAIDs in combination with anti-VEGF.

Study	Design, sample size, and study duration	NSAID	Treatment arms	Results	Author conclusions
Russo et al. (2013) [7]	Randomized, prospective, controlled, 56 eyes 6 months	Ketorolac 0.45% TID	Ketorolac plus IVR versus IVR alone for new exudative AMD	37.1 *µ*m greater CMT reduction at 6 months in ketorolac arm. No differences for VA or no. of injections	Topical ketorolac supplements the activity of intravitreal ranibizumab in reducing CMT in CNV
Gomi et al. (2012) [5]	Randomized, prospective, placebo-controlled, 30 eyes 6 months	Bromfenac 0.1% BID	Bromfenac plus IVR versus IVR alone for exudative AMD	Reduced CMT and fewer injections in bromfenac group, but similar VA	Bromfenac may reduce the frequency of IVR over 6 months in eyes with small CNV
Flaxel et al. (2012) [4]	Randomized, prospective, controlled, 30 eyes 12 months	Bromfenac 0.09% BID	Bromfenac plus IVR versus IVR alone for exudative AMD	63.3 *µ*m greater CMT reduction at 12 months in bromfenac arm. No differences for VA or no. of injections	Combination is efficacious for the treatment of exudative AMD
Chen et al. (2010) [32]	Retrospective, uncontrolled 25 eyes 3 months	Nepafenac 0.1% TID	Nepafenac plus IVR/IVB for recalcitrant exudative AMD	No changes in VA or CMT	No significant changes in VA or CMR, but a mild trend towards improved anatomy
Zweifel et al. (2009) [33]	Retrospective, uncontrolled 22 eyes 2 months	Bromfenac 0.09% BID	Bromfenac plus IVR/IVB for persistent exudative AMD	No changes in VA or CMT	No beneficial effect of adding bromfenac for persistent exudative AMD

AMD: age-related macular degeneration; CNV: choroidal neovascularization; VA: visual acuity; CMT: central macular thickness; IVR: intravitreal ranibizumab; IVB: intravitreal bevacizumab.
